# Joint binary response modelling for childhood comorbidity in Ethiopia

**DOI:** 10.1371/journal.pone.0268040

**Published:** 2022-05-18

**Authors:** Tesfaye Abera Bokoro, Habtamu Kiros Gebresilassie, Melkamu A. Zeru

**Affiliations:** 1 Department of Statistics, Haramaya University, Dire Dawa, Ethiopia; 2 Department of Statistics, Bahir Dar University, Bahir Dar, Ethiopia; Government College University Faisalabad, PAKISTAN

## Abstract

Childhood diarrhea and Acute Respiratory Infection (ARI) are two diseases with similar risk factors in tropical developing regions. The objective of this study was to employ a joint binary response model and identify risk factors for childhood diarrhea and ARI in children under the age of five. A joint binary response model that takes into account the interdependence of the two diseases was used. Explanatory variables such as residence, vaccination, mother’s education, and antenatal care visits during pregnancy were found to be statistically significant risk factors for diarrhea in the joint model, whereas residence, the number of children ever born, vaccination, mother’s education, and wealth index were found to be statistically significant risk factors for childhood Acute Respiratory Infection. We discovered a common odds ratio value (4.30) greater than one, indicating a positive relationship between the two childhood diseases. As a result, using a joint model to assess the risk factors for diarrhea and acute respiratory infection (ARI) was reasonable. Furthermore, the standard errors of the parameter estimates in the joint response model were found to be smaller than the corresponding standard errors in the separate models. The risk factors such as residence, vaccination, and mother’s education all had a significant effect on the two correlated dichotomous response variables, diarrhea and ARI.

## Introduction

Childhood Morbidity in low-income countries is commonly characterized by more than one health condition [1], and it is a challenge in many settings that can lead to death. On the other hand, coinfection due to overlapping risk factors such as nutrition, sanitation, and overcrowding [2] is extremely common among children under the age of five seeking care [3]. Diarrhea and ARI (Acute Respiratory Infection) are two diseases with common risk factors in tropical developing countries [4].

Diarrhea is one of the leading causes of death among Ethiopian children under the age of five. According to World Health Organization (WHO) estimates, diarrhea is responsible for more than 13% (one out of every ten) child deaths in Ethiopia [5]. According to previous research, the percentage of children under the age of five who had diarrhea decreased from 24 percent in 2000 to 12 percent in 2016 [6]. Furthermore, acute respiratory infection (ARI), particularly pneumonia, is one of the leading causes of morbidity and mortality in Ethiopia, accounting for 18% of all deaths [5]. The country has made a remarkable commitment to reducing ARI morbidity and mortality in children [7]. Deaths can be reduced if health interventions target one or more concurrent diseases [1]. Usually, childcare programs addressed single diseases such as diarrhea, acute respiratory infections, fever, and malaria infections [3]. Diarrhea and acute respiratory infections continue to be the leading causes of death in children under the age of five [8]. The illness of the two health conditions could be due to shared risk factors in childhood. For instance, diarrhea and acute respiratory diseases may share risk factors such as age as a child-dependent risk factor or poor sanitation and crowding as environmental risk factors [9].

Walker et al. [10] discovered that diarrhea and ARI have overlapping epidemiology, which is due in part to shared risk factors such as inadequate breastfeeding, malnutrition (which includes undernutrition, which is classified as underweight, stunting, and wasting), and a lack of zinc. According to Bbaale [4], breastfeeding for the first 6 months, socioeconomic status, type of house the child lived in, maternal occupation, child’s age, and nutritional status were all significant common factors associated with pneumonia and diarrhea.

Moreover, Leung et al. [11] found that younger age of child, male gender, undernutrition, lower maternal education, low wealth quintile, and poor breastfeeding practices were associated with the concurrent occurrence of diarrhea and pneumonia in under-five children in Dhaka. Hence, the majority of the risk factors for ARI and diarrhea are modifiable, since the two outcomes share some major risk factors, they can be addressed through integrated interventions [12].

Takele et al. [13] proposed a spatial effect model to explain the risk of diarrhea, cough, and fever comorbidity in Ethiopia and pointed out that childhood comorbidity is not distributed evenly across the country, with significant comorbidity hotspots in Tigray and Oromia. The findings suggest that using a combined morbidity management technique to reduce children’s illnesses and deaths has a significant impact. Regions with high comorbidity rates in children should be examined for healthcare interventions.

In order for childcare programs conducted by health institutions to be successful, statistical methods are required to assess the features of diseases that coexist. A recent trend in discovering similar patterns of variation has been signifying to employ a joint analysis that assesses risk factors for such diseases [14]. The joint modelling addresses the correlation of the two response variables with the same set of covariates. The two outcomes improve control over type I error rates in multiple tests and increase efficiency in parameter estimates.

Most previous health research in Ethiopia has focused on studying a single disease at a time. Separate analyses, however, may fail to provide a comprehensive picture of the epidemiology of the diseases and the combined effects of childhood diseases on the population under consideration due to common and overlapping risk factors [15]. A few studies on joint response modeling of correlated outcomes have concentrated on continuous, discrete, or a combination of discrete and continuous outcomes [16].

Epidemiological methodology and research have advanced over time, moving from studying a single disease to studying several diseases concurrently. Thus, in this paper, we focused on comorbidity in children under the age of five by employing a joint binary response model that takes into account the interdependence of the two infections when assessing risk factors. Hence, the Ethiopian government and other institutions involved in the health sector can use the findings of this study to improve the design and implementation of interventions that address the country’s exposure to multiple illnesses at an early age.

## Methodology

### Data source

The empirical analysis in this paper was based on data from 2016 Ethiopian Demographic and Health Survey (EDHS). The Demographic and Health Surveys (DHS) are a well-established source of reliable population data, with a strong emphasis on childhood diseases [6]. This study included 9917 under-five-year-old children, with each record containing information on childhood diseases as well as a list of covariates that may have an impact on children’s health.

### A description of the study variables

Let Y be a vector consisting of two dichotomous dependent variables, Y1 (Diarrhea Status) and Y2 (Acute Respiratory Infection Status). If the child had diarrhea and an acute respiratory infection, the two binary response variables would be set to ‘1’ or ‘0.’ The probabilities of occurrence of the possible joint outcomes of Y1 and Y2 for a set of m-paired observations can be illustrated as in Table 1.

**Table 1:** Combination of outcomes with corresponding probabilities of occurrence.

		**Acute Respiratory Infection (Y**_**2**_ **)**		**Total**
**Diarrhea (Y**_**1**_ **)**	y_1_ = 0	y_2_ = 0	y_2_ = 1	
		p_00_	p_01_	1-p_1_
	y_1_ = 1	p_10_	p_11_	p_1_
**Total**		1-p_2_	p_2_	1

Similarly, we can represent the joint and separate probabilities of Y_1_ and Y_2_ indicated in the Table 1 as, p_ij_ = P (y_1_ = i, y_2_ = j); i,j = 0,1 and p_k_ = P(y_k_ = 1), k = 1,2 respectively. Where:

**P**_**11**_ = the child has both diarrhea and ARI

**P**_**01**_ = the child has no diarrhea but has ARI

**P**_**10**_ = the child has diarrhea but not ARI

**P**_**00**_ = the child has neither diarrhea nor ARI

The following predictors were included in the final models: child’s age in months, child’s sex, residence, antenatal care visit, vaccination, mother’s education, number of children, and wealth index.

### Separate and joint models

The primary goal of the standard binary regression model is to predict the response variable categories as a function of the covariates in the model. However, in the bivariate logistic regression model, we attempt to investigate the relationship between two distinct dichotomous response variables (Y_1_ and Y_2_) by modelling them jointly as a function of the explanatory variables under consideration. Each pair of dependent variables (Y_i1_, Yi_2_) has four possible outcomes, as shown in Table 1. These are: (Y_i1_ = 1, Yi_2_ = 1), (Y_i1_ = 1, Yi_2_ = 0), (Y_i1_ = 0, Yi_2_ = 1), and (Y_i1_ = 0, Yi_2_ = 0).

The joint probability of each of these four outcomes was modelled using three systematic components, which are as follows:

■ The marginal (separate) distributions, that is *P*(Y_i1_ = 1) and *P*(Y_i2_ = 1)
■ The odds ratio, which is denoted by *ψ* describes the dependence of one marginal distribution on the other.

Bivariate logistic regression model or bivariate logistic odds-ratio model proposed by [17–19] is specified by modelling the marginal distribution of each of Y_j_, and the common odds ratio. The common odds ratio or ***ψ*** is defined as the ratio of the odds of Y_1_ = 1 given that Y_2_ = 1 and the odds of Y_1_ = 1 given that Y_2_ = 0, that is $\psi =\frac{p_{11}p_{00}}{p_{10}p_{01}}$. The odds ratio is used to measure the association between the two dichotomous response variables (Y_1_ and Y_2_); the value of ***ψ*** equal to one indicates a lack of association between Y_1_ and Y_2_.

Bivariate logistic regression provides adjusted estimates of concordance by simultaneously estimating covariate effects on the odds ratio, which describes the pair wise association structure. Furthermore, using bivariate logistic regression analysis may provide more precision than unadjusted estimates from other possible categorical distributions. The logistic regression models can be used to express the influence of the independent variables on the marginal probabilities, that is, the marginal probability of diarrhea (Y_1_) and ARI (Y_2_), as well as the odds ratio. Similarly, the relationship between covariates and each disease can be investigated using separate logistic regression models for each outcome:

$$logit[E(Y_{1j})]=logit[\mathrm{Pr}\left(Y_{1j}=1|X_{1j},\beta_{1}\right)]=\beta_{1}^{T}X_{1j}$$ (1)

$$logit[E(Y_{2j})]=logit[\mathrm{Pr}\left(Y_{2j}=1|X_{2j},\beta_{2}\right)]=\beta_{2}^{T}X_{2j}$$ (2)

These standard logistic regression models, as shown in Eqs (1) and (2), do not take into account the correlation between the two childhood diseases. However, we assume that the two diseases of the same individual share a set of common unobservable features. Let *u*_*j*_ denote the random intercept shared by the two diseases of the j^th^ (j = 1,2,…, m) individual. Let *ϕ*_1*i*_ and *ϕ*_2*i*_ define dummy variables with *ϕ*_1*i*_ =1 for i = 1 and *ϕ*_2*i*_ =1 for i = 2. Then, the joint response model for the bivariate logit is given by

$$\begin{array}{l} logit[E(Y_{ij}|u_{j})=logit[Pr(Y_{ij}=1|X_{ij},{\tilde{\beta }}_{i},u_{j})]\\ \;=\phi_{1j}({{\tilde{\beta }}_{1}}^{T}X_{1j}+u_{j})+\phi_{2j}({{\tilde{\beta }}_{2}}^{T}X_{2j}+u_{j}) \end{array}$$ (3)

As a result, in this equation, all individuals’ bivariate responses (Y_1j_,Y_2j_) are stacked into a single response vector. The random intercepts are assumed to differ from one individual to the next. Furthermore, the random intercepts are assumed to be normally distributed with a mean zero and variance $\sigma_{u}^{2}$. Let *G* denote the distribution of *u*_*j*_. Given the shared random intercept, an individual’s joint response is assumed to be independent. Using conditional independence from this assumption, we can write the joint response model’s likelihood function as follows:

$$\begin{array}{l} L(.)=\prod_{j=1}^{m}\mathrm{Pr}\left(Y_{ij}=1|X_{ij},{\tilde{\beta }}_{I},u_{j}\right)\\ \;=\prod_{j=1}^{m}\left\{\int Pr(Y_{ij}=1|X_{ij},{\tilde{\beta }}_{I},u_{j})\phi G(u_{j})\right\}\\ \;=\prod_{j=1}^{m}\left\{\int \prod_{i=1}^{2}Pr(Y_{ij}=1|X_{ij},{\tilde{\beta }}_{I},u_{j})\phi G(u_{j})\right\}\\ \;=\prod_{j=1}^{m}\left\{\int \prod_{i=1}^{2}\frac{e^{u_{j}+{{\tilde{\beta }}_{1}}^{T}X_{ij}}}{1+e^{u_{j}+{{\tilde{\beta }}_{1}}^{T}X_{ij}}}\phi G(u_{j})\right\} \end{array}$$ (4)

Following Ghebremichael [15], we used maximum likelihood estimation to obtain estimates of the model parameters (${\tilde{\beta }}_{1},{\tilde{\beta }}_{2},\sigma_{u}^{2}$). The estimation was based on maximizing the log-likelihood function, which is maximizing the logarithm of the likelihood function. However, because the integrals for Eq (4) do not have closed-form solutions, the parameters of the joint model were estimated simultaneously via maximum likelihood by evaluating the integrals using Gaussian adaptive quadrature approximation [20].

### Parameters in the joint and separate models

The parameters in the joint response model must be interpreted conditionally on the random intercepts, in contrast to the unconditional interpretations of the parameters in the separate models. The first set of parameters are conditional (subject-specific), while the second set of parameters are population-averaged (marginal). As a result, it’s impossible to compare the two sets of parameters directly. The marginal effects of the covariates in Eq (3) are required to compare the parameters in the joint response model to those obtained from the separate models. The subject-specific parameters of the joint model can be marginalised as described below. From the separate models (1)–(2), we can write the marginal mean as:

$$E(Y_{ij})=\frac{e^{\beta_{i}^{T}X_{ij}}}{1+e^{\beta_{i}^{T}X_{ij}}}\;,for\;i=1,2.$$ (5)

Similarly, in the joint response model (3), the conditional mean for the i^th^ response is given by:

$$E(Y_{ij}|b_{j})=\frac{e^{b_{j}+{\tilde{\beta }}_{i}^{T}X_{ij}}}{1+e^{b_{j}+{\tilde{\beta }}_{i}^{T}X_{ij}}}$$ (6)

The marginal information from the joint response model can be obtained by taking the expectation by the random intercept, as shown below:

$$\begin{array}{l} E(Y_{ij})=E(\{E(Y_{ij}|b_{j}\})\\ \;=E\left\{\frac{e^{b_{j}+{\tilde{\beta }}_{i}^{T}X_{ij}}}{1+e^{b_{j}+{\tilde{\beta }}_{i}^{T}X_{ij}}}\right\}\\ \;=\int_{-\infty }^{\infty }\frac{e^{b_{j}+{\tilde{\beta }}_{i}^{T}X_{ij}}}{1+e^{b_{j}+{\tilde{\beta }}_{i}^{T}X_{ij}}}dG(b_{j})\\ \;=\int_{-\infty }^{\infty }\frac{e^{b_{j}+{\tilde{\beta }}_{i}^{T}X_{ij}}}{1+e^{b_{j}+{\tilde{\beta }}_{i}^{T}X_{ij}}}\frac{1}{{\sqrt{2\pi }\sigma }_{b}}e^{{-b}_{j}^{2}/2\sigma_{b}^{2}}db_{j} \end{array}$$ (7)

We used an approximation of the marginal mean as in [15] because there is no exact closed-form solution for the marginal mean in the above equation. The magnitude of the between-subjects variation determines the discrepancies between the two parameters. When all individuals have the same random intercept value, the marginal parameters are roughly equivalent to the conditional parameters.

## Results and discussion

The analyses of this study were based on 9917 valid observations of children under the age of five in Ethiopia. As shown in Table 2, 5.9% and 8.2% of male child diarrhea and ARI cases, as well as 5.1% and 7.8% of female child diarrhea and ARI cases respectively, were reported in the previous fifteen days. Geographically, 4.6% of children with diarrhea and ARI lived in urban, compared to 22.1% who lived in rural areas. In terms of vaccination, 63.3% of children in Ethiopia were reported to have received various types of vaccination. The covariates residence, vaccination, antenatal visit, wealth index, and mother’s education were found to be significantly associated with diarrhea and ARI (P<0.05).

**Table 2:** Distribution of risk factors for childhood comorbidity in Ethiopia.

	Diarrhea N (%)			ARI N (%)		
	Yes	No	Sig.	Yes	No	Sig.
**Sex of child**						
Male	587 (5.9)	4476 (45.1)	0.05	811(8.2)	4252 (42.9)	0.855
Female	503 (5.1)	4352 (43.9)		771 (7.8)	4083 (41.2)	
**Place of residence**						
Urban	186(1.9)	1689 (16.9)	0.001	275 (2.7)	1601 (16.0)	0.001
Rural	904(9.0)	7137 (71.3)		1311 (13.1)	6736 (67.3)	
**Vaccination**						
Yes	2472 (9.1%)	2118 (54.2%)	0.001	2472(12.3%)	1993(51.0%)	0.002
No	1407(3.7%)	1264 (32.3%)		1407(5.4%)	1194(30.5%)	
**Antenatal visit**						
No	261 (3.8)	2054 (29.7)	0.001	384(5.6)	1933 (28.0)	0.029
Less 5 visits	445 (6.4)	2668 (38.6)		584 (8.4)	2531(36.6)	
More 5	211(3.1)	1227(17.7)		282 (4.1)	1156 (16.7)	
**Wealth index**						
Poorest	365 (3.6)	3311 (33.1)	0.001	514(5.1)	3163(31.6)	0.001
Porrer	192 (1.9)	1469 (14.7)		305 (3.0)	1359 (13.6)	
Middle	172 (1.7)	1206 (12.1)		228 (2.3)	1152 (11.5)	
Richer	159 (1.6)	1055 (10.5)		225 (2.2)	990(9.9)	
Richest	202 (2.0)	1785 (17.8)		314(3.1)	1673(16.7)	
**Mother’s Education**						
No	664 (6.6)	5679 (56.8)	0.001	982(9.8)	5366(53.6)	0.001
Primary	315 (3.1)	2188 (21.9)		456 (4.6)	2049 (20.5)	
Secondary	74(0.7)	613 (6.1)		101(1.0)	586(5.9)	
Higher	37(0.4)	346 (3.5)		47(0.5)	336(3.4)	

Table 3 shows that in the 2016 DHS, Ethiopia had a prevalence of 4.3% of children with diarrhea and ARI. On the other hand, 11.7% of the children only had ARI, and 6.7% of the children only had diarrhea morbidity.

**Table 3:** Cross-classification of children by diarrhea and ARI diseases in Ethiopia.

		Had ARI		Total
		No	Yes	
**Had diarrhea**	No	7671(77.4%)	1156(11.7%)	8827(89.0%)
	Yes	664(6.7%)	426(4.3%)	1090(11.0%)
Total		8335(84.0%)	1582(16.0%)	9917(100.0%)

χ^2^ = 488.65, df = 1, P<0.001.

As shown in Table 4, a common odds ratio (4.30) greater than one indicates a positive relationship between the two childhood diseases. As a result, the bivariate logistic regression model was deemed appropriate for investigating the risk factors associated with diarrhea and ARI diseases. This method provides estimates with vibrant concurrency properties by simultaneously estimating the effects of risk factors on the odds ratio. Furthermore, the model may provide better precision than unadjusted estimates obtained by considering a multinomial distribution. Regression equations are used to describe the effects of risk factors on the marginal probability of the two diseases and the odds ratio [21]. If Y_1_ and Y_2_ share some or all explanatory variables, and the effects of the shared explanatory variables are estimated jointly, the bivariate model is parametrically dependent. Covariates were screened before building the models because they showed a significant association with diarrhea and ARI in the chi-square and descriptive analyses.

**Table 4:** Results for risk factors of childhood diarrhea and ARI in separate and joint models.

Variable	Marginal Model						Joint Model					
	Diarrhea			ARI			Diarrhea			ARI		
	OR	SE	Sig.	OR	SE	Sig.	OR	SE	Sig.	OR	SE	Sig.
Constants	0.079	0.322	0.001	0.118	0.281	0.001	0.077	0.321	0.001	0.119	0.280	0.001
Resid1												
Resid2	1.287	0.246	0.307	1.730	0.218	0.012	1.287	0.247	0.307	1.724	0.217	0.013
Childsize	0.943	0.066	0.373	0.879	0.059	0.027	0.946	0.066	0.401	1.008	0.058	0.027
Vaccin1	1.391	0.117	0.005	1.306	0.101	0.008	1.410	0.116	0.003	1.303	0.100	0.008
Vaccin2												
Educ0												
Educ1	1.162	0.120	0.210	1.059	0.107	0.591	1.160	0.119	0.216	1.061	0.106	0.582
Educ2	0.611	0.285	0.014	0.601	0.244	0.037	0.604	0.284	0.027	0.600	0.243	0.036
Educ3	0.393	0.541	0.054	0.608	0.385	0.196	0.392	0.540	0.043	0.613	0.383	0.202
Wealth1												
Wealth2	1.066	0.142	0.653	1.208	0.124	0.126	1.084	0.141	0.568	1.207	0.123	0.128
Wealth3	1.163	0.152	0.319	1.082	0.138	0.567	1.163	0.151	0.320	1.082	0.138	0.570
Wealth4	.245	0.165	0.186	1.434	0.145	0.013	1.247	0.165	0.182	.432	0.145	0.013
Wealth5	.036	0.246	0.885	1.604	0.208	0.023	1.039	0.245	0.878	.599	0.207	0.024
Antenatal1	1.104	0.116	0.395	1.148	0.101	0.169	1.109	0.116	0.373	1.145	0.100	0.179
Antenatal2	1.585	0.156	0.003	1.200	0.145	0.207	1.611	0.156	0.002	1.194	0.144	0.221
Antenatal3												
							Odds ratio 4.30					

Maximum likelihood estimator of model parameters obtained in R using the VGAM and Zeligchoice packages from [22, 23]. As shown in Table 3, in the separate model, we found that covariates such as vaccination, mother’s education, and mother’s antenatal care were significantly associated with child diarrhea status, whereas residence, number of children, vaccination, mother’s education, and wealth index were significantly associated with childhood acute respiratory disease status. Standard models that ignore interdependence between two or more responses typically produce relatively biased estimates [15]. Thus, in assessing the potential risk factors for diarrhea and acute respiratory infection (ARI), we used a joint model that captures the dependence between childhood comorbidity.

Covariates such as vaccination, mother’s education, and antenatal care were found to be significant factors for childhood diarrhea in the joint response model. Similarly, among children under the age of five, residence, the number of children, vaccination, mother’s education, and wealth index were significant risk factors for acute respiratory infection (ARI). We noticed that children who did not receive vaccinations were 1.4 times more likely to develop diarrhea and ARI. In terms of parent education level, children from mothers with secondary and higher education were 0.60 and 0.39 times less likely to be affected by diarrhea, respectively, than children from mothers with no education. These findings are consistent with those of an Egyptian study [24]. Furthermore, children born to mothers who visited fewer antenatal care centers during their pregnancy were 1.61 times more likely to suffer from childhood diarrhea than children born to mothers who had regular antenatal care visits.

Children living in rural areas, on the other hand, were 1.72 times more likely than their counterparts to be infected with ARI disease. This could be due to a lack of healthcare and a poor way of life in rural areas. In terms of the number of children ever born, we concluded that as the number of children increases, so does the likelihood of ARI infection i.e. a child from a family with more children is more vulnerable to acute respiratory disease. Furthermore, children born to mothers with a secondary school education were 0.60 less likely to be infected with ARI than children born to mothers with no education. In terms of wealth quantile, we found that a child from a wealthy family was less likely to be infected by ARI, i.e. children from wealthier families were less likely to be infected by acute respiratory disease than children from poorer families. Being in a higher wealth status, as compared to the lowest (poorest), was found to reduce the likelihood of diarrhea and ARI, which is consistent with what was found in Uganda [4].

More specifically, we found that the standard errors of the parameter estimates in the joint response model were lower than the corresponding standard errors in the marginal models. This indicates that the joint model outperforms the marginal models in terms of efficiency. As a result of these findings, the need for jointly modeling two correlated responses was validated.

### Simulation result

Simulation can assist researchers in understanding the entire statistical model, making full use of parameter estimates, and communicating findings in a reader-friendly manner [25]. The means of the predicted probabilities of an event were calculated for each of the two categories of the dependent variable, as shown in S1–S5 Tables in S1 File. The absolute value of the difference between those two means was then calculated. If a model predicts well, cases with events should have high predicted values and cases without events should have low predicted values [26]. Along with the tables and graphs of the quantities of interest in S1 Fig in S1 File, the built joint model demonstrated its ability to make good predictions. The mean estimate for Y, the standard deviation, the median, and the 95% confidence interval measures of uncertainty around the average are all included in each row of statistics for expected and predicted values.

## Conclusion

In this paper, we focused on childhood comorbidity by employing a joint response model that takes into account the interdependence of the two diseases, diarrhea and ARI. Different socio-demographic and biological risk factors for diarrhea and ARI were considered. There was a 4.3 percent childhood comorbidity of diarrhea and ARI among 9917 under-five children from DHS (2016) in Ethiopia. There was a positive correlation between the two infections, which was taken into account in this study to obtain unbiased estimates. Previous research on diarrhea and ARI comorbidity modeled each disease separately, ignoring the possibility of a link between the two. The findings of this study contributed to the appreciation of joint models that deflate parameters that would otherwise be overestimated in separate models. Residence, vaccination, mother’s education, and antenatal care visits during pregnancy were found to be significant risk factors for diarrhea in the joint model, whereas residence, the number of children ever born, vaccination, mother’s education, and wealth index were statistically significant risk factors for ARI in childhood. The risk factors such as residence, vaccination, and mother’s education all had a significant impact on the two correlated binary response variables, diarrhea and ARI.

### Limitations

We did not take into account the spatial driving factors that contribute to geographic variations. Hence, future research should look into joint models for more than two diseases including spatial variation effect as well as other social and macroeconomic factors, in order to better explain variation in childhood multiple illnesses. Besides that, this study is limited to Ethiopia, limiting its applicability or generalizability to other countries. Furthermore, since cases were self-reported by family/caretakers and not subjected to clinical diagnosis, there might have been some recall bias and misclassification of diseases.

## Supporting information

S1 File (DOCX)
