# Supplementary material for: Joint binary response modelling for childhood comorbidity in Ethiopia
Source: PLoS One. 2022 May 18;17(5):e0268040. doi: 10.1371/journal.pone.0268040 (PMC9116622; doi:10.1371/journal.pone.0268040)
Supplement: S1 File — (DOCX) [file pone.0268040.s001.docx]

# Supporting Information

Quantities of interest from bivariate logit model

**S1 Table: Expected Values** **for x(lowest value of explanatory variable)**

mean sd 50% 2.5% 97.5%

Pr(Y1=0, Y2=0) 0.74854988 0.014409098 0.74913524 0.71984276 0.77557475

Pr(Y1=0, Y2=1) 0.12278475 0.010412208 0.12256883 0.10387570 0.14359428

Pr(Y1=1, Y2=0) 0.07540105 0.007805833 0.07522129 0.06004650 0.09160451

Pr(Y1=1, Y2=1) 0.05326432 0.006478022 0.05286303 0.04232368 0.06682350S2

**S2 Table: Predicted Values for x(lowest value of explanatory variable)**

0 1

(Y1=0, Y2=0) 0.289 0.711

(Y1=0, Y2=1) 0.843 0.157

(Y1=1, Y2=0) 0.888 0.112

(Y1=1, Y2=1) 0.980 0.020

**S3 Table: Expected Values for x_1_(highest value of the explanatory variable)**

mean sd 50% 2.5% 97.5%

Pr(Y1=0, Y2=0) 0.83784969 0.04727187 0.84443911 0.729413394 0.9124655

Pr(Y1=0, Y2=1) 0.10031222 0.03759618 0.09337025 0.046248931 0.1913946

Pr(Y1=1, Y2=0) 0.04132368 0.02283666 0.03550673 0.011918510 0.1019103

Pr(Y1=1, Y2=1) 0.02051441 0.01307836 0.01775972 0.005519475 0.0505012

**S4 Table: Predicted Values for x_1_(highest value of the explanatory variable)**

0 1

(Y1=0, Y2=0) 0.160 0.840

(Y1=0, Y2=1) 0.897 0.103

(Y1=1, Y2=0) 0.951 0.049

(Y1=1, Y2=1) 0.992 0.008

**S5 Table: Fist Difference**

mean sd 50% 2.5% 97.5%

Pr(Y1=0,Y2=0) 0.08929981 0.04562680 0.09516639 -0.01514334 0.16144689

Pr(Y1=0,Y2=1) -0.02247253 0.03606915-0.02729431 -0.07446998 0.06537456

Pr(Y1=1,Y2=0) -0.03407737 0.02257006 0.03796583 -0.06516763 0.02111796

Pr(Y1=1,Y2=1) -0.03274992 0.01293345-0.03455233 -0.05159941-0.00274422

**S1 Fig 1: Graphs of quantities of interest for Diarrhea model (a), ARI model (b) and Joint model(c)**

1.
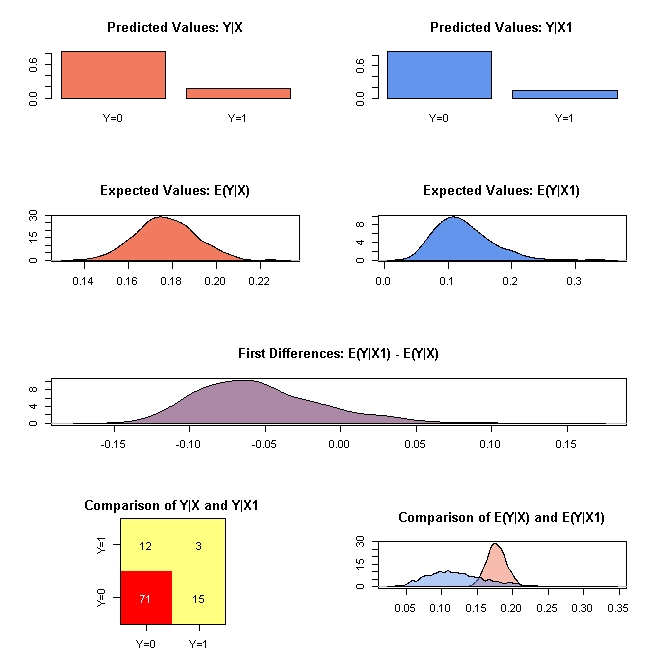

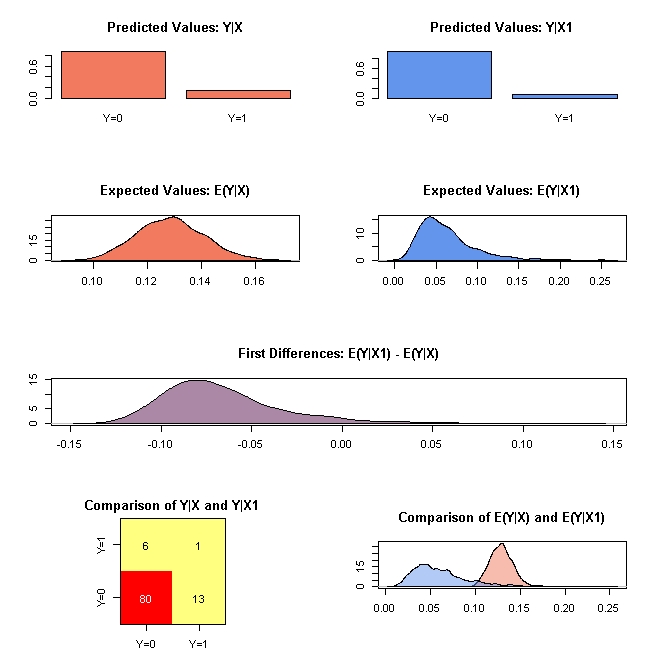
 (b)


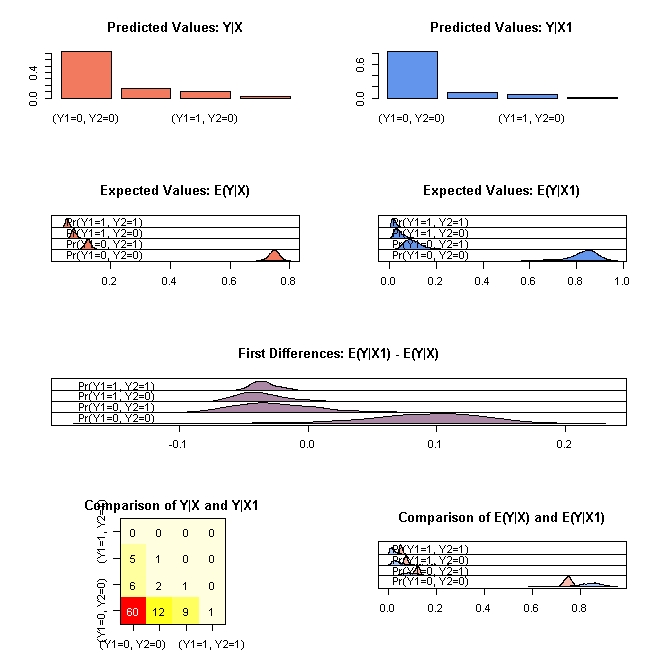
 (c)
